# Association between dermatoglyphic patterns and growth patterns of subjects with skeletal class I relation: A cross sectional study

**DOI:** 10.12688/f1000research.121961.2

**Published:** 2022-07-04

**Authors:** Keerthan Shashidhar, Kuttappa M N, U S Krishna Nayak, Neevan D'Souza, Mahabalesh Shetty, Sonika Achalli

**Affiliations:** 1 Orthodontics and Dentofacial Orthopedics, A B Shetty Memorial Institute of Dental Sciences; Nitte (Deemed to be University), Mangalore, Karnataka, 575018, India; 2 Department of Biostatistics, K.S Hegde Medical Academy; , Nitte (Deemed to be University), Mangalore, Karnataka, 575018, India; 3 Department of Forensic Medicine, K.S Hegde Medical Academy; Nitte (Deemed to be University), Mangalore, Karnataka, 575018, India; 4 Oral Medicine and Radiology, A B Shetty Memorial Institute of Dental Sciences; Nitte (Deemed to be University), Mangalore, Karnataka, 575018, India

**Keywords:** Dermatoglyphics, Orthodontics, Mandible, Growth, Orthodontics, Preventive

## Abstract

**Background:** To assess the relationship between dermatoglyphic patterns and various growth patterns of the mandible.

**Methods:** Patients with Class I Skeletal relation were selected after clinical diagnosis followed by digitally tracing the cephalogram. The patients were subdivided into three groups of mandibular divergence patterns ie Average, Horizontal and Vertical. 90 samples ie 30 in each group were selected for the study. The fingerprints of all the selected subjects were then extracted digitally and analysed for the most dominant pattern in each hand.

**Results:** For the left hand, there was a statistically significant (P<0.05) association between fingerprint pattern and growth pattern when Horizontal growers were compared to Average and Vertical Growers. For the right hand, there was a statistically significant (P<0.05) association between fingerprint pattern and growth pattern when Horizontal growers were compared to Average Growers. A significant association (P<0.05) between fingerprint pattern and growth pattern was also found when average growers were compared to vertical growers.

**Conclusions:** Horizontal growers had 80% frequency of appearance of whorls in their left hand and 67% in their right hand. Horizontal growers could easily be differentiated from the average and vertical growers because of the dominance of whorl pattern in their hands.

Composite and arch pattern were more frequent in vertical growers when compared to horizontal and average growers.

## Introduction

The craniofacial growth determines the type of shape of the head, shape of the face and the presence or absence of any anomalies in the head and face region. Many factors influence the craniofacial growth which ultimately maps the face of an individual.

One of the factors that contributes a large role in determining the final outcome of the face is genetics. The human mandible continues to grow even after the maxilla attains its final position. It is because of this reason that the mandibular growth pattern cannot be easily predicted. Facial growth relative to a cranial base line proceeds along a vector composed of variable amounts of horizontal (forward) growth or vertical (downward) growth. ^1^ These growth patterns of the mandible are each associated with varied treatment modalities in the orthodontic field. The rotations of the mandible can occur for a variety of reasons, but there is undeniably an intrinsically determinant factor ie the genes that play a role in the establishment of the pattern of growth of the lower jaw. ^2^

Recently, a lot of interest has been shown towards dermatoglyphics in the dental fraternity. It has also been reported that dermatoglyphics is associated with a number of medical conditions. The interest of dermatoglyphics in medicine was generated when abnormal dermal patterns were observed in Down’s syndrome. ^3^ Dermatoglyphic patterns have also been shown to be related to oral clefts, ^4^ dental arch forms, ^5^ dental caries, ^6^ carcinoma of the breast, ^7^ Type 2 diabetes, hypertension ^8^ and head and neck cancer. ^9^

Since dermatoglyphic patterns develop intrauterine (12 ^th^–24 ^th^ week) during the same period as the development of the mandible (14 ^th^–29 ^th^ week) and genetics plays a determining factor in their development, it can be hypothesized that they bear relationship with each other. Since it is said that the dermal configurations remain constant throughout life except for overall size, ^10^^,^^11^ fingerprint patterns and other details of dermal ridges could offer distinct advantages wherein, they could be used as a screening tool, which is easily accessible, economical and may serve as non-invasive marker to detect early malocclusion.

In the field of orthodontics, many studies have been conducted to assess the relationship between dermatoglyphic patterns and sagittal malocclusion. However, currently, only two studies ^12^^,^^13^ have focused on the relationship between dermatoglyphic patterns and growth patterns of individuals. Both these studies had low sample sizes and had not clearly defined the parameters of the study.

Hence, the objective of this study was to assess the relationship of dermatoglyphic patterns with diverging growth patterns of individuals with Class I Skeletal Relationship with the hypothesis that there was a relationship between dermatoglyphic patterns and diverging growth patterns.

## Methods

This cross-sectional study was conducted in A B Shetty Memorial Institute of Dental Sciences, Karnataka, India after obtaining ethical clearance from the institutional ethical committee (Ethical Clearance No. ABSM/ETH/2020-18/092).

Systemically healthy dental patients with no missing teeth (except third molars) between the ages of 20 and 35 years who attended the department of orthodontics and dentofacial orthopaedics at A B Shetty Dental College from December 2020 until December 2021 without any history of previous orthodontic treatment were recruited for the study. Nonprobability convenience sampling was used to select the samples that fit into the inclusion criteria. A detailed case history was taken of each patient to rule out patients with history of habits, or history of any surgical procedures on the digits of the hand and face. Patients with Class I Skeletal relation were selected after clinical diagnosis followed by digitally tracing the cephalogram. Based on the Cephalometric values, the patients were subdivided into three groups of mandibular divergence patterns ie Average, Horizontal and Vertical. Based on 5% level of significance, 80% power, effect size of 0.39 and degree of freedom 6, the total samples required were 90. 111 samples were selected for this study, of which 21 samples were eliminated due to conflicting cephalometric values. Finally, the 90 samples ie 30 in each group were selected for the study. Fingerprints of the subjects were recorded digitally for each finger and then analysed. All subjects signed a written consent form indicating their approval to participate in this study. This human observational study manuscript conforms to STROBE guidelines for cross-sectional studies.

### Cephalometric evaluation

Skeletal Class I relation was diagnosed by assessing the ANB angle (2±2), the Beta Angle (27-35) and the Wits Analysis (0±1).

The growth pattern for an individual was diagnosed by assessing the FMA angle, the SNGoGn Angle and the Jarabacks ratio. For the Average group the FMA ranged from 25±5, the SNGoGn ranged from 32±4 and the Jarabacks ratio ranged from 62-65. These values were used as the norms to diagnose the Average group of mandibular divergence. Any values below the range of FMA, SNGoGn and above the Jarabacks ratio would be classified as Horizontal Growth Pattern. Any values above the norms of FMA, SNGoGn and below the Jarabacks ratio would be classified as Vertical Growth Pattern.

All cephalograms were traced digitally on the One Ceph software by an experienced orthodontist. The same orthodontist traced each cephalogram 10 days after it was first traced. This was done to avoid any intra-operator errors.

### Dermatoglyphics

**Figure 1.**
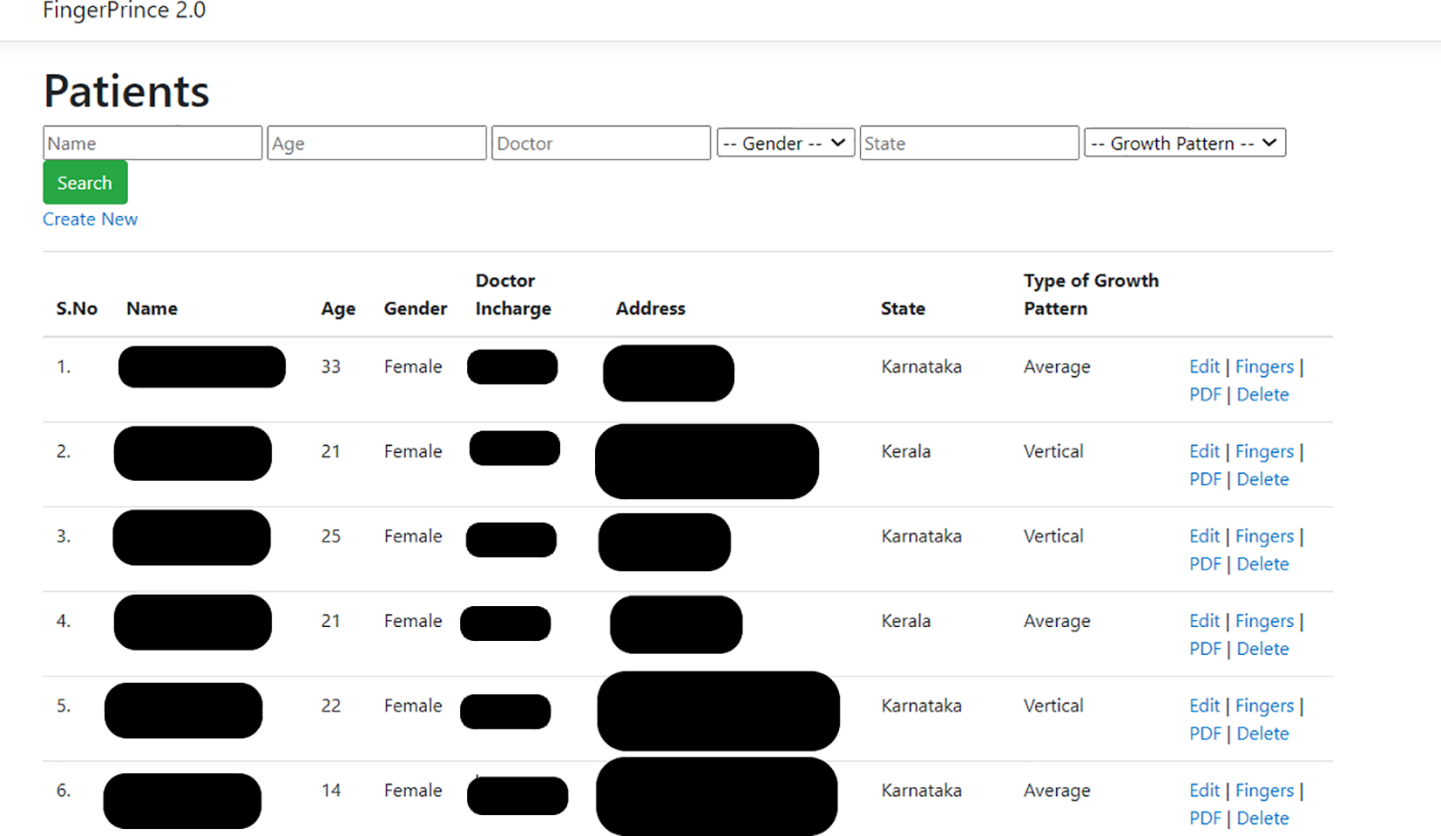
Index of subjects data as seen in the ‘Fingerprince’ application.

**Figure 2.**
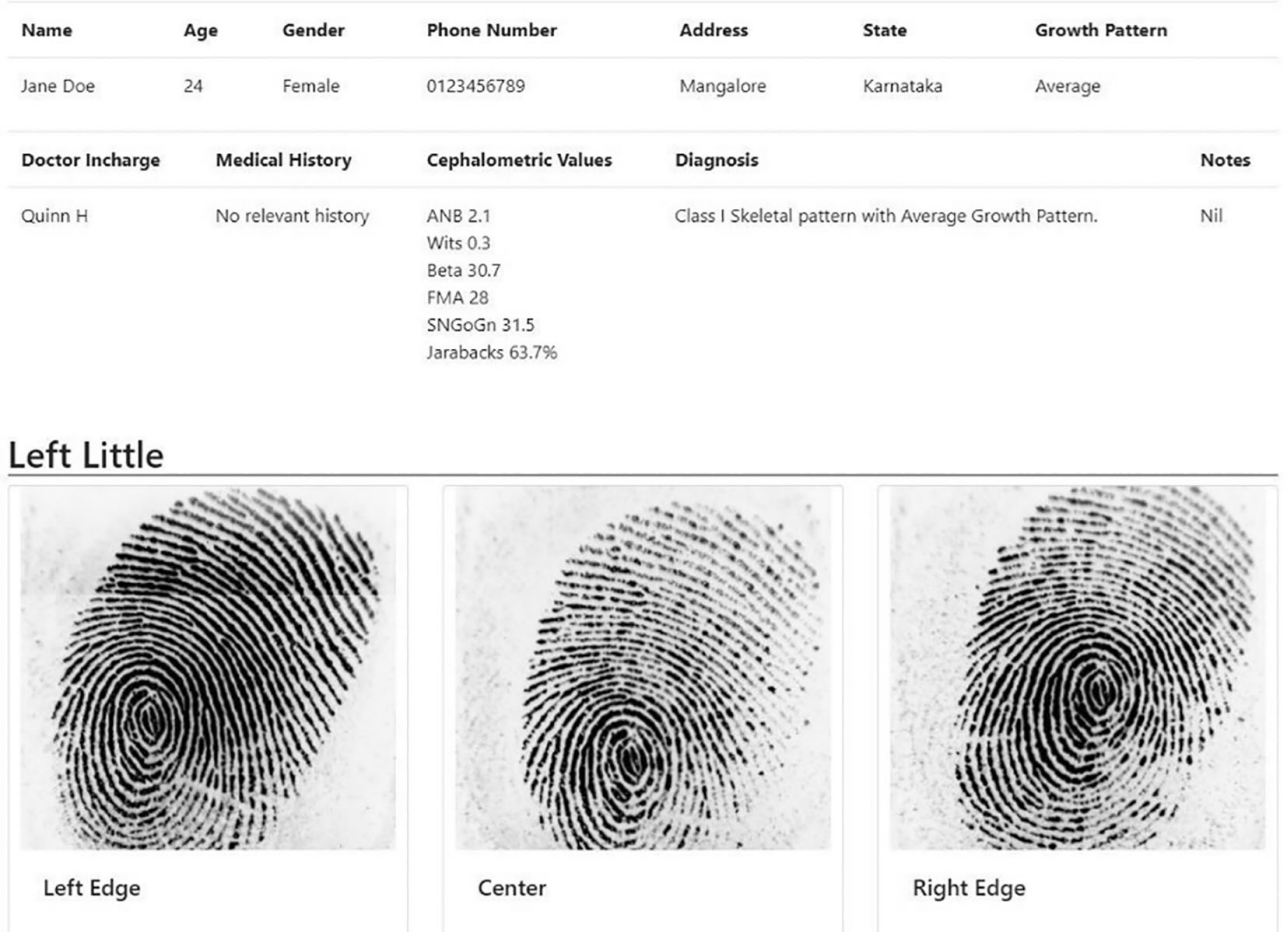
Data of a sample patient as seen in the ‘Fingerprince’ application after collection of fingerprints.

**Figure 3.**
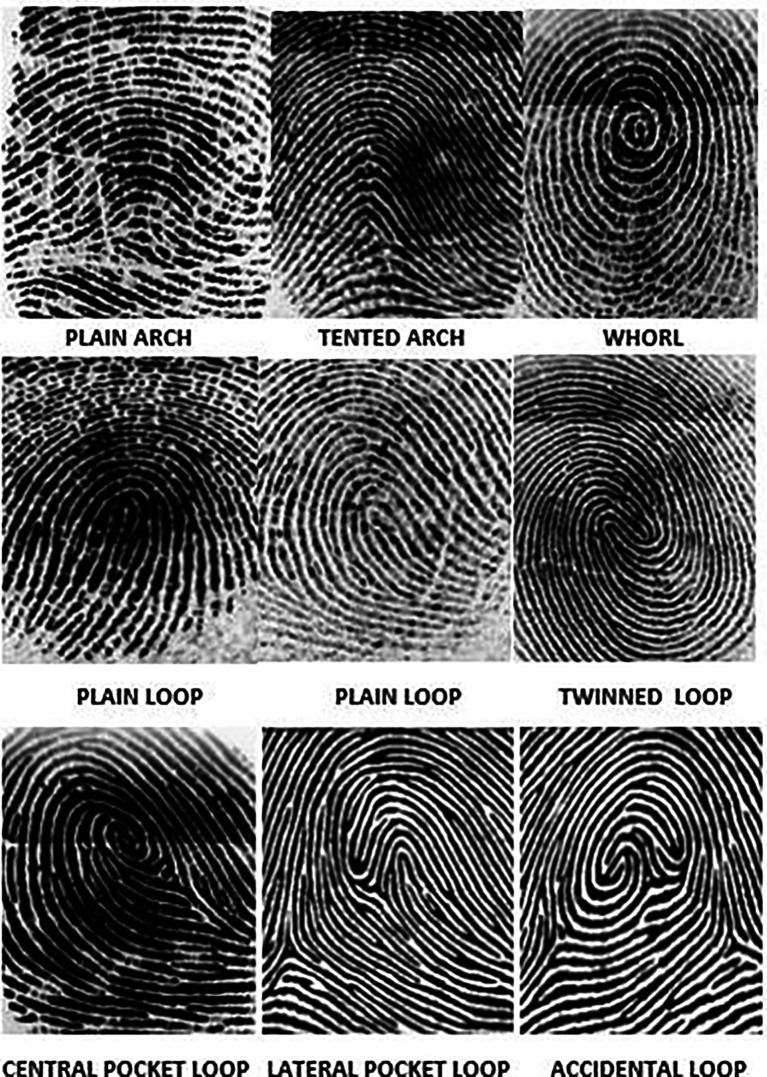
Henry’s classification of dermatoglyphic patterns.

The DG patterns of patients were recorded for all 10 digits of the hands using a digital fingerprint scanner MFS100 (Mantra Tech v54/v54OTG). The subject was asked to wash his/her hand with soap and water, followed by which his/her hand was allowed to be air dried. Once the fingers were dry, the fingers were scanned using the digital scanner. A custom designed application for this study called ‘Fingerprince’ (Designed in Arizona, United States of America) ( Figures 1, 2) was used to store and analyse the fingerprint of the subjects. The application also helped store the patient’s case history as well as the cephalometric values. Henry’s classification was used to classify the fingerprints into loops, whorl, arch and composite patterns ( Figure 3).

Loops consisted of radial loops and ulnar loops. Whorls consisted of Plain Whorl, Double loop whorl, accidental whorl and central pocket whorl. Arches consisted of plain arches and tented arches. Composite patterns consisted of central pocket loop whorl, lateral pocket loops and accidental loops. In cases where there was no dominant fingerprint pattern, it was classified as a Tie between the 2 patterns.

### Statistical analysis

The results were evaluated using Version 20 of the Statistical package for social sciences (SPSS INC, Chicago, Illinois, USA). Fisher's Exact test was used to find the association between growth pattern and dermatoglyphic patterns, where P<0.05 was considered significant.

## Results

The results of the Left hand are shown in Tables 1- 4 and the results of the Right hand are shown in Tables 5- 8.

**Table 1.** Overall comparison for between groups (Left hand).

			Left dominant					Total
			Arch	C	Loop	Tie	Whorl	
Growth pattern	Average	Count	0	1	28	1	0	30
		% within Growth pattern	0.0%	3.3%	93.3%	3.3%	0.0%	100.0%
	Horizontal	Count	0	0	5	1	24	30
		% within Growth pattern	0.0%	0.0%	16.7%	3.3%	80.0%	100.0%
	Vertical	Count	1	2	21	3	3	30
		% within Growth pattern	3.3%	6.7%	70.0%	10.0%	10.0%	100.0%

**Table 2.** The Fisher’s exact test between the average and horizontal groups (Left hand).

	Dermatoglyphic patterns		C	Loop	Tie	Whorl	Total
Growth pattern	Average	Count	1	28	1	0	30
		% within Growth pattern	3.3%	93.3%	3.3%	0.0%	100.0%
	Horizontal	Count	0	5	1	24	30
		% within Growth pattern	0.0%	16.7%	3.3%	80.0%	100.0%

Fishers value: 47.855; P value: 0.000.

It shows a value of 47.855 and a P value of <0.05, indicating an association between the Growth patterns and Fingerprint patterns (P<0.05).

**Table 3.** The Fisher’s exact test between the average and vertical groups (Left hand).

	Dermatoglyphic patterns		Arch	C	Loop	Tie	Whorl	Total
Growth pattern	Average	Count	0	1	28	1	0	30
		% within Growth pattern	0.0%	3.3%	93.3%	3.3%	0.0%	100.0%
	Vertical	Count	1	2	21	3	3	30
		% within Growth pattern	3.3%	6.7%	70.0%	10.0%	10.0%	100.0%

Fishers value: 5.875; P value: 0.142.

It shows a value of 5.875 and a P value of 0.142, indicating no association between the Growth patterns and Fingerprint patterns (P>0.05).

**Table 4.** The Fisher’s exact test between the horizontal and vertical groups (Left hand).

	Dermatoglyphic patterns		Arch	C	Loop	Tie	Whorl	Total
Growth pattern	Horizontal	Count	0	0	5	1	24	30
		% within Growth pattern	0.0%	0.0%	16.7%	3.3%	80.0%	100.0%
	Vertical	Count	1	2	21	3	3	30
		% within Growth pattern	3.3%	6.7%	70.0%	10.0%	10.0%	100.0%

Fishers value: 31.355; P value: .000.

It shows a value of 31.355 and a P value < 0.05, indicating an association between the Growth patterns and Fingerprint patterns (P<0.05).

**Table 5.** Overall comparison for between groups (Right hand).

			Right dominant					Total
			Arch	C	Loop	Tie	Whorl	
Growth pattern	Average	Count	0	0	28	2	0	30
		% within Growth pattern	0.0%	0.0%	93.3%	6.7%	0.0%	100.0%
	Horizontal	Count	0	0	8	2	20	30
		% within Growth pattern	0.0%	0.0%	26.7%	6.7%	66.7%	100.0%
	Vertical	Count	1	0	21	6	3	30
		% within Growth pattern	3.3%	0.0%	66.7%	20.0%	10.0%	100.0%

**Table 6.** The Fisher’s exact test between the average and horizontal groups (Right hand).

	Dermatoglyphic pattern		Arch	C	Loop	Tie	Whorl	Total
Growth pattern	Average	Count	0	0	28	2	0	30
		% within Growth pattern	0.0%	0.0%	93.3%	6.7%	0.0%	100.0%
	Horizontal	Count	0	0	8	2	20	30
		% within Growth pattern	0.0%	0.0%	26.7%	6.7%	66.7%	100.0%

Fishers value: 35.815; P value: 0.000.

It shows a value of 35.815 and a P value of < 0.05, indicating an association between the Growth patterns and Fingerprint patterns (P<0.05).

**Table 7.** The Fisher’s exact test between the average and vertical groups (Right hand).

	Dermatoglyphic pattern		Arch	C	Loop	Tie	Whorl	Total
Growth pattern	Average	Count	0	0	28	2	0	30
		% within Growth pattern	0.0%	0.0%	93.3%	6.7%	0.0%	100.0%
	Vertical	Count	1	0	20	6	3	30
		% within Growth pattern	3.3%	0%	66.7	20%	10%	100.0%

Fishers value: 6.752; P value: 0.031.

It shows a value of 6.752 and a P value of 0.031, indicating an association between the Growth patterns and Fingerprint patterns (P<0.05).

**Table 8.** The Fisher's exact test between horizontal and vertical groups (Right hand).

	Dermatoglyphic pattern		Arch	C	Loop	Tie	Whorl	Total
Growth pattern	Horizontal	Count	0	0	8	2	20	30
		% within Growth pattern	0.0%	0.0%	26.7%	6.7%	66.7%	100.0%
	Vertical	Count	1	0	20	6	3	30
		% within Growth pattern	3.3%	0%	66.7	20%	10%	100.0%

Fishers Value: 21.294; P value: 0.001.

It shows a value of 21.294 and a P value of 0.001, indicating an association between the Growth patterns and Fingerprint patterns (p<0.05).

### Left hand

With respect to the left hand, average growers had the loop pattern as the most dominant pattern with a frequency percentage of 93.3%, followed by composite pattern with a frequency percentage of 3.3% and there were 3.3% of the population that showed a tie between two patterns ( Table 1).

Horizontal growers had the whorl pattern as the most dominant pattern with a frequency percentage of 80%, followed by loop pattern with a frequency percentage of 16.7% and there were 3.3% of the population that showed a tie between two patterns ( Table 1). The exceptional dominance of the whorl pattern in the horizontal group is striking since the average and vertical groups have only 1/8 ^th^ the amount of whorls than the horizontal group.

Vertical growers had the loop pattern as the most dominant pattern with a frequency percentage of 70%, followed by whorl pattern with a frequency percentage of 10%, followed by the composite pattern with a frequency percentage of 6.7% and lastly the arch pattern with a frequency percentage of 3%. In this group there were 10% of the population that showed a tie between two patterns ( Table 1). The higher incidence of presence of composite pattern and arch pattern in the vertical group was an important finding.

Upon intergroup comparison between the average group and the horizontal group ( Table 2), there was a statistically significant association between fingerprints and growth patterns (P<0.05).

Comparison between average group and vertical group ( Table 3) showed no statistically significant association between fingerprints and growth patterns (P>0.05).

Comparison between the horizontal group and the vertical group ( Table 4) showed a statistically significant association between fingerprints and growth patterns (P<0.05).

### Right hand

With respect to the right hand, average growers had the loop pattern as the most dominant pattern with a frequency percentage of 93.3%. There were 6.7% of the population that showed a tie between two patterns ( Table 5).

Horizontal growers had the whorl pattern as the most dominant pattern with a frequency percentage of 66.6%, followed by loop pattern with a frequency percentage of 26.7% and there were 6.7% of the population that showed a tie between two patterns ( Table 5). Although reduced when compared to the left hand, the frequency of the whorl pattern in the horizontal group is still striking since the average and vertical groups have only 1/6 ^th^ the amount of whorls than the horizontal group.

Vertical growers had the loop pattern as the most dominant pattern with a frequency percentage of 66.7%, followed by whorl pattern with a frequency percentage of 10%, followed by the composite pattern with a frequency percentage of 6.7% and lastly the arch pattern with a frequency percentage of 3.3%. In this group there were 20% of the population that showed a tie between two patterns ( Table 5). The higher incidence of presence of composite pattern and arch pattern in the vertical group.

Upon intergroup comparison between the average group and the horizontal group ( Table 6), there was a statistically significant association between fingerprints and growth patterns (P<0.05).

Comparison between average group and vertical group ( Table 7) also showed a statistically significant association between finger prints and growth patterns (P<0.05).

Comparison between the horizontal group and the vertical group ( Table 8) showed a statistically significant association between finger prints and growth patterns (P<0.05).

A point to be remembered is that we only chose the most dominant pattern in each hand (Appearing at least three times in each hand). Therefore, even though none of the growth patterns had a dominant composite pattern in the right hand, it does not mean that the composite pattern did not appear in the right hand.

## Discussion

Being able to predict what one’s facial pattern would be like by assessing their fingerprints may seem far-fetched. But the results of this study prove otherwise. Dermatoglyphics has shown to be positively associated with cleft lip and palate. Some authors also claim that they are able to predict dental malocclusion as well. However, all studies so far seem to have conflicting results. While one may say that the loop pattern is dominant in class I malocclusion, ^14^ another may say that it is the whorl pattern. ^15^ Sagittal skeletal relations have also been studied. The results here also seem to be contrasting. While one author says that Arches ^16^ are the most common pattern in skeletal class I relation, others say it is the loop pattern ^17^^,^^18^

Only two studies have tried to find a relation between growth patterns and dermatoglyphics.

While Nivedita Sahoo ^12^ found that there was an increased incidence of whorls in the horizontal group and loops in the vertical group (which is similar to the results found in the present study), the study failed to have a detailed inclusion criteria for the selection of subjects. The skeletal relation of the subjects hadn’t been mentioned and the average group pattern had not been studied. Both of these shortcomings have been addressed in the present study.

A recent study by Harmeet *et al.* ^13^ showed a higher presence of loops in skeletal class I subjects but concluded saying that there was no statistically significant association between dermatoglyphics and various growth pattern. However, a point to be noted is that their sample size consisted of only 15 subjects in each of the three groups, while the present study had double the sample size of their study.

While both the studies mentioned above used the ink method to extract the fingerprints, we used the digital method to extract the fingerprints. We found this method to have an easier mode of operation, better ease of convenience and higher accuracy than the ink method/lipstick method. A recent study by Loveday *et al.* ^19^ has proven that the digital method of collecting fingerprints was the easiest and the most user friendly methods when compared to the ink/lipstick method. The present study also involved the use of a custom made software called ‘Fingerprince’ which helped store the Case history of the subjects and their fingerprints.

The present study included subjects with purely class I skeletal relation, with the sole objective of finding out if there was any relation of dermatoglyphics with the normal skeletal relation. However, we did categorize the Skeletal Class I relation into three categories ie the average, horizontal and vertical growth pattern groups. The present study shows that the loop pattern was dominant in both the average and the vertical growth pattern group. But it contradicts other studies in the Class I Horizontal Group. Despite having a Class I skeletal relation, more than 73% of the horizontal growers had the whorl pattern as a dominant pattern making it a very important discovery.

This shows that horizontal growers could easily be identified by seeing which pattern was dominant in both their hands. The present study showed that horizontal growers had 80% frequency of appearance of whorls in their left hand and 67% in their right hand.

This could mean that when a child is born, and if he/she has a dominance of whorl pattern on their fingers, we could predict that the child may have a horizontal growth pattern.

Another important discovery was the increased incidence of finding arches and composite patterns in the vertical growers when compared to the average and horizontal group. Although it was a clinical difference and not a statistically significant difference found while assessing the subjects, it does help in understanding the relationship of dermatoglyphics with growth patterns. We also observed that the average growth pattern had 93% frequency of appearance of loops and a negligible percentage of whorls and composite pattern.

While we can confirm and say that horizontal group of patients can easily be differentiated from average and vertical growers, the same cannot be said for average and vertical growers.

A higher sample size will be required to see if the difference between the average and vertical groups are statistically significant.

The results of this study have drastic implications in treatment planning and diagnosis. For example, if we are able to identify a child with prints that show a dominance of whorl pattern, we can predict that the child may have a horizontal growth pattern. This can be easily intercepted using cervical headgears or anterior bite planes to bring about an average growth pattern. Since orthodontic treatment modalities change according to the growth pattern, even vertical growers can be intercepted to try and achieve an average growth pattern.

A problem we faced was the conflicting cephalometric values that made a subject borderline class I/II, or Average-Horizontal, Average to Vertical. The authors took a decision to eliminate such samples and therefore reduced the samples from 111 to 90. Hence the samples had True Class I Skeletal Relation, true average growth pattern, true horizontal growth pattern and true certical growth pattern ( Table 9).

**Table 9.** Average values for all parameters in all 3 groups.

Parameter	Average Growth pattern	Horizontal Growth pattern	Vertical Growth pattern
ANB (Degrees)	2.4	2.6	3.1
WITS (mm)	0.4	0.3	0.02
BETA (Degrees)	32.1	30.7	32.6
FMA (Degrees)	27.8	23.4	33.1
SNGoGn (Degrees)	31.7	23.8	37.1
Jarabacks (%)	64.3	71	60.5

Although the sample size was higher than other studies, we do feel that a drawback of the present study was the low sample size. A higher sample size with a target population of a specific area would help us understand the demographic and/or ethnic variation of the dermatoglyphics (if any) and also help validate the findings of the present study. Another drawback of the study was that we only used Henry’s classification where he broadly classified it into loops, arches, whorls and composites. A study focussing on its sub classifications would provide a more in-depth detail on the type of fingerprints that were dominant. While this study focused on finding the dominant pattern in each hand, it would be interesting to note if there was any particular finger which showed a consistent pattern for each growth pattern. Since the dermatoglyphic patterns and the lower jaw form during the same embryologic period, and the current study shows that there is some kind of association between the two, further genetic studies could be conducted to isolate a particular gene responsible for the same. Having found interesting results for this study, the above mentioned points can increase the scope of research in this field.

While all the current methods to predict the growth of the mandible are cumbersome, technique sensitive and manual, predicting the growth pattern by analysing the fingerprints seems to be the most easiest, cost-effective, non-invasive method and can be done anytime and anywhere. The only prerequisite would be to have knowledge of the different types of dermatoglyphic patterns.

## Conclusion

The following conclusions can be made from this study

1) Horizontal growers had the highest incidence of whorl pattern as the dominant pattern in both the left and right hands when compared to average growers and horizontal growers.
2) Average growers had the highest incidence of loop pattern as the dominant pattern in both the left and right hands when compared to average growers and horizontal growers.
3) The presence of Arch patterns and composite patterns (although not dominant) were more common in vertical growers than the horizontal and average growers.

## Data availability

### Underlying data

The images of the fingerprints cannot be shared because of ethical issues since it can be tied to the identity of a person. However, the interpretation of the data is available in Excel format.

Open Science Framework: Association between dermatoglyphic patterns and growth patterns of subjects with skeletal class I relation: A Cross Sectional Study, https://doi.org/10.17605/OSF.IO/5VFKJ.

Data are available under the terms of the Creative Commons Attribution 4.0 International license (CC-BY 4.0).
