# Supplementary material for: Association between dermatoglyphic patterns and growth patterns of subjects with skeletal class I relation: A cross sectional study
Source: F1000Res. 2022 Jul 4;11:597. Originally published 2022 Jun 1. [Version 2] doi: 10.12688/f1000research.121961.2 (PMC9214274; doi:10.12688/f1000research.121961.2)
Supplement: Peer review history

# Peer review: Reviewer response for version 2

**DOI:** 10.5256/f1000research.135598.r143179

**Published:** 2022-07-05

**Authors:** Gautham Sivamurthy

**Affiliations:** 1 Department of Orthodontics, School of Dentistry, University of Dundee, Dundee, Scotland, UK

Is the work clearly and accurately presented and does it cite the current literature?

Yes

If applicable, is the statistical analysis and its interpretation appropriate?

Yes

Are all the source data underlying the results available to ensure full reproducibility?

Yes

Is the study design appropriate and is the work technically sound?

Yes

Are the conclusions drawn adequately supported by the results?

Yes

Are sufficient details of methods and analysis provided to allow replication by others?

Yes

Reviewer Expertise:

Clinical trials in orthodontics assessin growht modification appliances for Class II skeletal patterns, arch wire materials and deep bite correction, cephalometrics and its clinical use.

I confirm that I have read this submission and believe that I have an appropriate level of expertise to confirm that it is of an acceptable scientific standard.

# Peer review: Reviewer response for version 2

**DOI:** 10.5256/f1000research.135598.r143178

**Published:** 2022-07-05

**Authors:** Masanobu Abe

**Affiliations:** 1 Division for Health Service Promotion, The University of Tokyo, Tokyo, Japan

The manuscript has been appropriately revised and there are no further corrections to be made.

Is the work clearly and accurately presented and does it cite the current literature?

Yes

If applicable, is the statistical analysis and its interpretation appropriate?

Yes

Are all the source data underlying the results available to ensure full reproducibility?

Yes

Is the study design appropriate and is the work technically sound?

Yes

Are the conclusions drawn adequately supported by the results?

Yes

Are sufficient details of methods and analysis provided to allow replication by others?

Yes

Reviewer Expertise:

Oral Malignancies, Malocclusion, Periodontal diseases

I confirm that I have read this submission and believe that I have an appropriate level of expertise to confirm that it is of an acceptable scientific standard.

# Peer review: Reviewer response for version 1

**DOI:** 10.5256/f1000research.133883.r139573

**Published:** 2022-06-21

**Authors:** Masanobu Abe

**Affiliations:** 1 Division for Health Service Promotion, The University of Tokyo, Tokyo, Japan

Authors assessed the relationship between dermatoglyphic patterns and various growth patterns of the mandible and they found close association between dermatoglyphic patterns and diverging growth patterns of the mandible. The results suggest that mandibular development may be predicted by other physical characteristics. The research is substantial and the manuscript is well written. The results would contribute to the future development of orthodontics.

- The sample size and statistical tests are appropriate for this analysis.
- The results are well summarized. 
- The discussion is well written. However, following issues (#1 and #2 ) need to be discussed.
  - **#1:** The authors found interesting results regarding the relationship between skin pattern and mandibular growth patterns, possibly the scientific mechanisms for the relationship need to be discussed.
  - **#2:** The authors state that one of the factors influencing final outcome of the face is genetics in the Introduction. It would be interesting whether facial finish and skin pattern formation are directly related, involving the same genes (e.g., growth-related genes), or whether they are indirect events. I would appreciate discussion on this point.
- The conclusion is appropriate, but authors have to describe the limitations of their study.
- The references are considered to be adequate.

Is the work clearly and accurately presented and does it cite the current literature?

Yes

If applicable, is the statistical analysis and its interpretation appropriate?

Yes

Are all the source data underlying the results available to ensure full reproducibility?

Yes

Is the study design appropriate and is the work technically sound?

Yes

Are the conclusions drawn adequately supported by the results?

Yes

Are sufficient details of methods and analysis provided to allow replication by others?

Yes

Reviewer Expertise:

Oral Malignancies, Malocclusion, Periodontal diseases

I confirm that I have read this submission and believe that I have an appropriate level of expertise to confirm that it is of an acceptable scientific standard.

# Response

**Published:** 2022-06-27

**Authors:** Keerthan Shashidhar

**Affiliations:** A B Shetty Memorial Institute of Dental Sciences, India

Dear Dr. Abe. M​

 We would like to thank you for taking time off your hectic schedule to critically analyze and ​​​​​​review our study. We have gone through your suggestions and would like to respond to the same. Your comments are labeled as Reviewer 2 and it will be followed by our Comments.

**Reviewer 2:** *"The authors found interesting results regarding the relationship between skin pattern and mandibular growth patterns, possibly the scientific mechanisms for the relationship need to be discussed."*

**Comment from Authors :** Yes, we understand what you mean and have now included a paragraph in the discussion for the same. However, the only 'scientific' mechanism that we can deduce this to is the fact that the dermatoglyphic prints and the lower jaw form during the same embryologic period. Having found this link, we believe that this opens scope for further research to solidify the claim.

**Reviewer 2:** *"The authors state that one of the factors influencing final outcome of the face is genetics in the Introduction. It would be interesting whether facial finish and skin pattern formation are directly related, involving the same genes (e.g., growth-related genes), or whether they are indirect events. I would appreciate discussion on this point."*

**Comment from Authors :** Yes, Exactly. Till date, we do not have any study that has focused to find a gene that is responsible for both the fingerprints and the lower jaw growth. We believe that this study forms the base for future genetic studies to find the exact link. Thank you for pointing this out. We have now included a line on the same in our discussion.

**Reviewer 2:** *"The conclusion is appropriate, but authors have to describe the limitations of their study."*

**Comment from Authors : **We have now included the drawbacks of the study.

# Peer review: Reviewer response for version 1

**DOI:** 10.5256/f1000research.133883.r139572

**Published:** 2022-06-20

**Authors:** Gautham Sivamurthy

**Affiliations:** 1 Department of Orthodontics, School of Dentistry, University of Dundee, Dundee, Scotland, UK

Interesting piece of work which would add to the evidence base in this field. Work clearly presented with appropriate study design used and useful to our clinical practice as many a times it is difficult to predict growth pattern of the mandible.

- Good sample size for analysis, which could have been strengthened with a sample size calculation.
- Robust Cephalometric evaluation done - possible typo 'Angles Class I malocclusion was diagnosed by assessing the ANB angle (2±2).....', which should have been 'Skeletal Class I....'
- Good information regarding methodology to replicate the study, along appropriate use of software to analyse cephalograms and finger prints. Possibly information on intraexaminer reliability on cephalogram tracing could have been included.
- Appropriate statistical test employed. - but a very low p value (<0.001) has been used to test significance? - possible typo maybe and meant to be <0.05?
- Results well discussed and appropriate data provided in the tables. By 'tie' does the authors mean 'correlation'? If so, authors to consider use of this word instead?
- Conclusion drawn adequately, but since the population studied is presumably from south India and Class I skeletal, hence the authors should consider including this in the conclusion statement?
- Adequate references used - could reference number 9 kindly be checked as article relates to dentistry and reference quoted is to support correlation between dermatoglyphics and cancer?

Overall a well conducted study and well written article which is appropriate for indexing.

Is the work clearly and accurately presented and does it cite the current literature?

Yes

If applicable, is the statistical analysis and its interpretation appropriate?

Yes

Are all the source data underlying the results available to ensure full reproducibility?

Yes

Is the study design appropriate and is the work technically sound?

Yes

Are the conclusions drawn adequately supported by the results?

Yes

Are sufficient details of methods and analysis provided to allow replication by others?

Yes

Reviewer Expertise:

Clinical trials in orthodontics assessin growht modification appliances for Class II skeletal patterns, arch wire materials and deep bite correction, cephalometrics and its clinical use.

I confirm that I have read this submission and believe that I have an appropriate level of expertise to confirm that it is of an acceptable scientific standard.

# Response

**Published:** 2022-06-27

**Authors:** Keerthan Shashidhar

**Affiliations:** A B Shetty Memorial Institute of Dental Sciences, India

Dear Dr. Gautham S, 

 We would like to thank you for taking time off your hectic schedule for critically analysing and reviewing our article. We have gone through the points that you have put forth and would like to explain ourselves accordingly. Each of your point has been mentioned below, followed by our comment.

- **Reviewer 1**: *"Good sample size for analysis, which could have been strengthened with a sample size calculation."*
**Comment from Authors :** Yes, we agree. We have now added the Sample Size calculations to the study.
- **Reviewer 1**: *"Robust Cephalometric evaluation done - possible typo 'Angles Class I malocclusion was diagnosed by assessing the ANB angle (2±2).....', which should have been 'Skeletal Class I....'"*
**Comment from Authors : **Yes, we apologize for the typo. The same has been corrected in the manuscript.
- **Reviewer 1**: *"Good information regarding methodology to replicate the study, along appropriate use of software to analyse cephalograms and finger prints. Possibly information on intraexaminer reliability on cephalogram tracing could have been included."*
**Comment from Authors :** The tracings were done by an experienced orthodontist. Each cephalogram was traced twice to avoid any intra-operator errors. We have added this point in the manuscript as well.
- **Reviewer 1: *"*** *Appropriate statistical test employed. - but a very low p value (<0.001) has been used to test significance? - possible typo maybe and meant to be <0.05?"*
**Comment from Authors :** Yes, we apologize for the typo. Thank you for pointing it out. Changes have been made in the manuscript.
- **Reviewer 1**: *"Results well discussed and appropriate data provided in the tables. By 'tie' does the authors mean 'correlation'? If so, authors to consider use of this word instead?"*
**Comment from Authors : **By 'Tie' we mean, that we could not find a Dominant pattern in the hand. For example, if 2 fingers showed a whorl pattern, 2 showed a loop pattern and 1 showed a composite pattern, then it would be considered a tie between loop and whorl and would come under the 'Tie' Category. Hope it is understood now.
- **Reviewer 1**: *"Conclusion drawn adequately, but since the population studied is presumably from south India and Class I skeletal, hence the authors should consider including this in the conclusion statement?"*
**Comment from Authors : **Yes, we understand what you mean. While the majority of the subjects were from south India, we did not prioritize the need to make it a population study. Hence, some subjects were from other parts of India as well. A population study would require a different methodology and we did not intend to make this a population-based study.
- **Reviewer 1: *"*** *Adequate references used - could reference number 9 kindly be checked as article relates to dentistry and the reference quoted is to support correlation between dermatoglyphics and cancer?"*
**Comment from Authors : **Yes, we apologize for the mistake. we quoted the review article instead of quoting the source article. The source article has now been quoted. Thank you for pointing it out.
